# Prevention and Control of Seasonal Influenza with Vaccines: Recommendations of the Advisory Committee on Immunization Practices — United States, 2020–21 Influenza Season

**DOI:** 10.15585/mmwr.rr6908a1

**Published:** 2020-08-21

**Authors:** Lisa A. Grohskopf, Elif Alyanak, Karen R. Broder, Lenee H. Blanton, Alicia M. Fry, Daniel B. Jernigan, Robert L. Atmar

**Affiliations:** ^1^Influenza Division, National Center for Immunization and Respiratory Diseases, CDC; ^2^Battelle Memorial Institute, Atlanta, Georgia; ^3^Immunization Safety Office, National Center for Emerging and Zoonotic Infectious Diseases, CDC; ^4^Baylor College of Medicine, Houston, Texas

## Abstract

This report updates the 2019–20 recommendations of the Advisory Committee on Immunization Practices (ACIP) regarding the use of seasonal influenza vaccines in the United States (*MMWR Recomm Rep 2019;68[No. RR-3]*). Routine annual influenza vaccination is recommended for all persons aged ≥6 months who do not have contraindications. For each recipient, a licensed and age-appropriate vaccine should be used. Inactivated influenza vaccines (IIVs), recombinant influenza vaccine (RIV4), and live attenuated influenza vaccine (LAIV4) are expected to be available. Most influenza vaccines available for the 2020–21 season will be quadrivalent, with the exception of MF59-adjuvanted IIV, which is expected to be available in both quadrivalent and trivalent formulations.

Updates to the recommendations described in this report reflect discussions during public meetings of ACIP held on October 23, 2019; February 26, 2020; and June 24, 2020. Primary updates to this report include the following two items. First, the composition of 2020–21 U.S. influenza vaccines includes updates to the influenza A(H1N1)pdm09, influenza A(H3N2), and influenza B/Victoria lineage components. Second, recent licensures of two new influenza vaccines, Fluzone High-Dose Quadrivalent and Fluad Quadrivalent, are discussed. Both new vaccines are licensed for persons aged ≥65 years. Additional changes include updated discussion of contraindications and precautions to influenza vaccination and the accompanying Table, updated discussion concerning use of LAIV4 in the setting of influenza antiviral medication use, and updated recommendations concerning vaccination of persons with egg allergy who receive either cell culture–based IIV4 (ccIIV4) or RIV4.

The 2020–21 influenza season will coincide with the continued or recurrent circulation of SARS-CoV-2 (the novel coronavirus associated with coronavirus disease 2019 [COVID-19]). Influenza vaccination of persons aged ≥6 months to reduce prevalence of illness caused by influenza will reduce symptoms that might be confused with those of COVID-19. Prevention of and reduction in the severity of influenza illness and reduction of outpatient illnesses, hospitalizations, and intensive care unit admissions through influenza vaccination also could alleviate stress on the U.S. health care system. Guidance for vaccine planning during the pandemic is available at https://www.cdc.gov/vaccines/pandemic-guidance/index.html.

This report focuses on recommendations for the use of vaccines for the prevention and control of seasonal influenza during the 2020–21 season in the United States. A brief summary of the recommendations and a link to the most recent Background Document containing additional information are available at https://www.cdc.gov/vaccines/hcp/acip-recs/vacc-specific/flu.html. These recommendations apply to U.S.-licensed influenza vaccines used within Food and Drug Administration (FDA)–licensed indications. Updates and other information are available from CDC’s influenza website (https://www.cdc.gov/flu). Vaccination and health care providers should check this site periodically for additional information.

## Introduction

Influenza viruses typically circulate in the United States annually, most commonly from the late fall through the early spring. Most persons who become ill with influenza virus infection recover without serious complications or sequelae. However, influenza can be associated with serious illnesses, hospitalizations, and deaths, particularly among older adults, very young children, pregnant women, and persons of all ages with certain chronic medical conditions (*1*–*7*). Influenza also is an important cause of missed work and school (*8*–*10*). Routine annual influenza vaccination for all persons aged ≥6 months who do not have contraindications has been recommended by CDC and CDC’s Advisory Committee on Immunization Practices (ACIP) since 2010 (*11*).

The effectiveness of influenza vaccination varies depending on several factors, such as the age and health of the recipient; the type of vaccine administered; the types, subtypes (for influenza A), and lineages (for influenza B) of circulating influenza viruses; and the degree of similarity between circulating viruses and those included in the vaccine (*12*). However, vaccination provides important protection from influenza illness and its potential complications. During the six influenza seasons from 2010–11 through 2015–16, influenza vaccination prevented an estimated 1.6–6.7 million illnesses, 790,000–3.1 million outpatient medical visits, 39,000–87,000 hospitalizations, and 3,000–10,000 respiratory and circulatory deaths each season in the United States (*13*). During the recent severe 2017–18 season, notable for an unusually long duration of widespread high influenza activity throughout the United States and higher rates of outpatient visits and hospitalizations compared with recent seasons, vaccination prevented an estimated 7.1 million illnesses, 3.7 million medical visits, 109,000 hospitalizations, and 8,000 deaths (*14*), despite an overall estimated vaccine effectiveness of 38% (62% against influenza A[H1N1]pdm09 viruses, 22% against influenza A[H3N2] viruses, and 50% against influenza B viruses).

In late 2019, a novel coronavirus, SARS-CoV-2, emerged as a cause of severe respiratory illness (*15*). In March 2020, the World Health Organization (WHO) declared coronavirus disease 2019 (COVID-19) a global pandemic (*16*). The common signs and symptoms of COVID-19 (e.g., fever, cough, and dyspnea) (*17*) can also occur with influenza illness. As of August 2020, SARS-CoV-2 continues to circulate and cause severe illness in the United States and worldwide. The extent to which SARS-CoV-2 will circulate over the course of the 2020–21 influenza season is unknown. However, during the continued or recurrent circulation of SARS-CoV-2 concurrently with influenza viruses during the upcoming fall and winter, influenza vaccination of persons aged ≥6 months can reduce prevalence of illness caused by influenza, and can also reduce symptoms that might be confused with those of COVID-19. Prevention of and reduction in the severity of influenza illness and reduction of outpatient illnesses, hospitalizations, and intensive care unit admissions through influenza vaccination also could alleviate stress on the U.S. health care system. Guidance for vaccine planning during the pandemic is available at https://www.cdc.gov/vaccines/pandemic-guidance/index.html. 

This report updates the 2019–20 ACIP recommendations regarding the use of seasonal influenza vaccines (*18*) and provides recommendations and guidance for vaccine providers regarding the use of influenza vaccines in the United States for the 2020–21 season. Various formulations of influenza vaccines are available (Table 1). Contraindications and precautions for the use of influenza vaccines are summarized (Table 2). Abbreviations are used in this report to denote the various types of vaccines (Box).

**TABLE 1:** Influenza vaccines — United States, 2020–21 influenza season*

Trade name (Manufacturer)	Presentation	Age indication	HA (IIVs and RIV4) or virus count (LAIV4) for each vaccine virus (per dose )	Route	Mercury (from thimerosal) *µ*g/0.5mL
**IIV4**					
Standard dose, egg based^†^					
Afluria Quadrivalent (Seqirus)	0.25-mL PFS^§^	6 through 35 mos	7.5 *µ*g/0.25 mL 15 *µ*g/0.5 mL	IM^¶^	—
	0.5-mL PFS	≥3 yrs			—
	5.0-mL MDV^§^	≥6 mos (needle/syringe) 18 through 64 yrs (jet injector)			24.5
Fluarix Quadrivalent (GlaxoSmithKline)	0.5-mL PFS	≥6 mos	15 *µ*g/0.5 mL	IM^¶^	—
FluLaval Quadrivalent (GlaxoSmithKline)	0.5-mL PFS	≥6 mos	15 *µ*g/0.5 mL	IM^¶^	—
Fluzone Quadrivalent (Sanofi Pasteur)	0.5-mL PFS**	≥6 mos	15 *µ*g/0.5 mL	IM^¶^	—
	0.5-mL SDV	≥6 mos			—
	5.0-mL MDV	≥6 mos			25
Standard dose, cell culture based (ccIIV4)					
Flucelvax Quadrivalent (Seqirus)	0.5-mL PFS	≥4 yrs	15 *µ*g/0.5 mL	IM^¶^	—
	5.0-mL MDV	≥4 yrs			25
High dose, egg based^†^ (HD-IIV4)					
Fluzone High-Dose Quadrivalent (Sanofi Pasteur)	0.7-mL PFS	≥65 yrs	60 *µ*g/0.7 mL	IM^¶^	—
Standard dose, egg based^†^ with MF59 adjuvant (aIIV4**)**					
Fluad Quadrivalent (Seqirus)	0.5-mL PFS	≥65 yrs	15 *µ*g/0.5 mL	IM^¶^	—
**IIV3 **					
Standard dose, egg based^†^ with MF59 adjuvant (aIIV3)					
Fluad (Seqirus)	0.5-mL PFS	≥65 yrs	15 *µ*g/0.5 mL	IM^¶^	—
**RIV4**					
Recombinant HA					
Flublok Quadrivalent (Sanofi Pasteur)	0.5-mL PFS	≥18 yrs	45 *µ*g/0.5 mL	IM^¶^	—
**LAIV4 **					
Egg based^†^					
FluMist Quadrivalent (AstraZeneca)	0.2-mL prefilled single-use intranasal sprayer	2 through 49 yrs	10^6.5-7.5^ fluorescent focus units/0.2 mL	NAS	—

**Abbreviations:** ACIP = Advisory Committee on Immunization Practices; FDA = Food and Drug Administration; HA = hemagglutinin; IIV3 = inactivated influenza vaccine, trivalent; IIV4 = inactivated influenza vaccine, quadrivalent; IM = intramuscular; LAIV4 = live attenuated influenza vaccine, quadrivalent; MDV = multidose vial; NAS = intranasal; PFS = prefilled syringe; RIV4 = recombinant influenza vaccine, quadrivalent; SDV = single-dose vial.

* Vaccination providers should consult FDA-approved prescribing information for 2020–21 influenza vaccines for the most complete and updated information, including (but not limited to) indications, contraindications, warnings, and precautions. Package inserts for U.S.-licensed vaccines are available at https://www.fda.gov/vaccines-blood-biologics/approved-products/vaccines-licensed-use-united-states. Availability and characteristics of specific products and presentations might change and/or differ from what is described in this table and in the text of this report.

^†^ History of severe allergic reaction (e.g., anaphylaxis) to egg is a labeled contraindication to the use of most IIVs and LAIV4. However, ACIP recommends that persons with a history of egg allergy may receive any licensed, recommended influenza vaccine that is otherwise appropriate for their age and health status. Those who report having had reactions to egg involving symptoms other than urticaria (e.g., angioedema or swelling, respiratory distress, lightheadedness, or recurrent emesis) or who required epinephrine or another emergency medical intervention should be vaccinated in an inpatient or outpatient medical setting (including, but not necessarily limited to, hospitals, clinics, health departments, and physician offices) supervised by a health care provider who is able to recognize and manage severe allergic reactions, if a vaccine other than ccIIV4 or RIV4 is used.

^§^ The dose volume for Afluria Quadrivalent is 0.25 mL for children aged 6 through 35 months and 0.5 mL for persons aged ≥3 years.

^¶^ IM-administered influenza vaccines should be given by needle and syringe only, with the exception of the MDV presentation of Afluria Quadrivalent, which may alternatively be given by the PharmaJet Stratis jet injector for persons aged 18 through 64 years only. For adults and older children, the recommended site for intramuscular influenza vaccination is the deltoid muscle. The preferred site for infants and young children is the anterolateral aspect of the thigh. Additional guidance regarding site selection and needle length for intramuscular administration is available in the ACIP General Best Practice Guidelines for Immunization, available at https://www.cdc.gov/vaccines/hcp/acip-recs/general-recs/downloads/general-recs.pdf.

** Fluzone Quadrivalent is currently licensed for ages 6 through 35 months at either 0.25 mL or 0.5 mL per dose; however, 0.25-mL prefilled syringes are not expected to be available for the 2020–21 influenza season. If a prefilled syringe of Fluzone Quadrivalent is used for a child in this age group, the dose volume will be 0.5mL per dose.

**TABLE 2:** Contraindications and precautions for the use of influenza vaccines — United States, 2020–21 influenza season*

Vaccine type	Contraindications	Precautions
IIV3 and IIV4	History of severe allergic reaction to any component of the vaccine,^†^ or to a previous dose of any influenza vaccine	Moderate or severe acute illness with or without fever History of Guillain-Barré syndrome within 6 weeks of receipt of influenza vaccine
RIV4	History of severe allergic reaction to any component of the vaccine	Moderate or severe acute illness with or without fever History of Guillain-Barré syndrome within 6 weeks of receipt of influenza vaccine
LAIV4	History of severe allergic reaction to any component of the vaccine^†^ or to a previous dose of any influenza vaccine^§^ Concomitant aspirin- or salicylate-containing therapy in children and adolescents^§^ Children aged 2 through 4 years who have received a diagnosis of asthma or whose parents or caregivers report that a health care provider has told them during the preceding 12 months that their child had wheezing or asthma or whose medical record indicates a wheezing episode has occurred during the preceding 12 months Children and adults who are immunocompromised due to any cause, including but not limited to immunosuppression caused by medications, congenital or acquired immunodeficiency states, HIV infection, anatomic asplenia, or functional asplenia (e.g., due to sickle-cell anemia) Close contacts and caregivers of severely immunosuppressed persons who require a protected environment Pregnancy Persons with active communication between the CSF and the oropharynx, nasopharynx, nose, or ear or any other cranial CSF leak Persons with cochlear implants^¶^ Receipt of influenza antiviral medication within the previous 48 hours for oseltamivir and zanamivir, previous 5 days for peramivir, and previous 17 days for baloxavir**	Moderate or severe acute illness with or without fever History of Guillain-Barré syndrome within 6 weeks of receipt of influenza vaccine Asthma in persons aged ≥5 years Other underlying medical conditions that might predispose to complications after wild-type influenza infection (e.g., chronic pulmonary, cardiovascular [except isolated hypertension], renal, hepatic, neurologic, hematologic, or metabolic disorders [including diabetes mellitus])

**Abbreviations:** ACIP = Advisory Committee on Immunization Practices; ccIIV4 = cell culture–based inactivated influenza vaccine; CSF = cerebrospinal fluid; FDA = Food and Drug Administration; IIV3 = inactivated influenza vaccine, trivalent; IIV4 = inactivated influenza vaccine, quadrivalent; LAIV4 = live-attenuated influenza vaccine, quadrivalent; RIV4 = recombinant influenza vaccine, quadrivalent.

* Vaccination providers should check FDA-approved prescribing information for 2020–21 influenza vaccines for the most complete and updated information, including (but not limited to) indications, contraindications, warnings, and precautions. Package inserts for U.S.-licensed vaccines are available at https://www.fda.gov/vaccines-blood-biologics/approved-products/vaccines-licensed-use-united-states.

^†^ History of severe allergic reaction (e.g., anaphylaxis) to egg is a labeled contraindication to the use of most IIVs and LAIV4. However, ACIP recommends that persons with a history of egg allergy may receive any licensed, recommended influenza vaccine that is otherwise appropriate for their age and health status. Those who report having had reactions to egg involving symptoms other than urticaria (e.g., angioedema or swelling, respiratory distress, lightheadedness, or recurrent emesis) or who required epinephrine or another emergency medical intervention should be vaccinated in an inpatient or outpatient medical setting (including, but not necessarily limited to, hospitals, clinics, health departments, and physician offices), supervised by a health care provider who is able to recognize and manage severe allergic reactions, if a vaccine other than ccIIV4 or RIV4 is used.

^§^ Labeled contraindication noted in package insert.

^¶^ Age-appropriate injectable vaccines are recommended for persons with cochlear implant due to the potential for CSF leak, which might exist for some period after implantation. Providers might consider consultation with a specialist concerning risk for persistent CSF leak if an age-appropriate inactivated or recombinant vaccine cannot be used.

** Use of LAIV4 in context of influenza antivirals has not been studied; however, interference with activity of LAIV4 is biologically plausible, and this possibility is noted in the package insert for LAIV4. In the absence of data supporting an adequate minimum interval between influenza antiviral use and LAIV4 administration, the intervals provided are based on the half-life of each antiviral. The interval between influenza antiviral receipt and LAIV4 for which interference might potentially occur might be further prolonged in the presence of medical conditions that delay medication clearance (e.g., renal insufficiency). Influenza antivirals might also interfere with LAIV4 if initiated within 2 weeks after vaccination. Persons who receive antivirals during the period starting with the specified time before receipt of LAIV4 through two weeks after receipt of LAIV4 should be revaccinated with an age-appropriate IIV or RIV4.

BOX **Abbreviation conventions for vaccines discussed in this report**

- Main influenza vaccine types include:
  - **IIV** = Inactivated Influenza Vaccine
  - **RIV** = Recombinant Influenza Vaccine
  - **LAIV** = Live Attenuated Influenza Vaccine
- Numerals following letter abbreviations indicate the number of influenza virus hemagglutinin (HA) antigens represented in the vaccine:
  - **4** for quadrivalent vaccines: one A(H1N1), one A(H3N2), and two B viruses (one from each lineage)
  - **3** for trivalent vaccines: one A(H1N1), one A(H3N2), and one B virus (from one lineage)
- Prefixes are used when necessary to refer to some specific inactivated vaccine types:
  - **a** for adjuvanted inactivated influenza vaccine (aIIV3 and aIIV4)
  - **cc** for cell culture–based inactivated influenza vaccine (ccIIV4)
  - **HD** for high-dose inactivated influenza vaccine (HD-IIV4)
  - **SD** for standard-dose inactivated influenza vaccine (SD-IIV4)

This report focuses on recommendations and guidance for the use of seasonal influenza vaccines for the prevention and control of influenza in the United States. A summary of these recommendations and a Background Document containing additional information on influenza, influenza-associated illness, and influenza vaccines are available at https://www.cdc.gov/vaccines/hcp/acip-recs/vacc-specific/flu.html.

## Methods

ACIP provides annual recommendations for the use of influenza vaccines for the prevention and control of influenza in the United States. The ACIP Influenza Work Group meets by teleconference once to twice per month throughout the year. Work group membership includes several voting members of ACIP, representatives of ACIP Liaison Organizations, and consultants. Discussions include topics such as influenza surveillance, vaccine effectiveness and safety, vaccination coverage, program feasibility, cost-effectiveness, and vaccine supply. Presentations are requested from invited experts, and published and unpublished data are discussed.

The Background Document that supplements this report is updated periodically to reflect recent additions to the literature related to recommendations made in previous seasons and minor changes in guidance for the use of influenza vaccines (e.g., guidance for timing of vaccination and other programmatic issues, guidance for dosage in specific populations, guidance for selection of vaccines for specific populations that are already recommended for vaccination, and changes that reflect use that is consistent with indications and prescribing information licensed by the Food and Drug Administration [FDA]). The summary included in the Background Document for such topics is not a systematic review; it is intended to provide an overview of current literature, with updated articles being identified primarily through a broad search for English-language articles on influenza and influenza vaccines. In general, systematic review and evaluation of evidence using the Grading of Recommendations Assessment, Development and Evaluation (GRADE) approach (*19*) is performed for new recommendations or substantial changes in the recommendations (e.g., expansion of the recommendation for influenza vaccination to new populations not previously recommended for vaccination or potential preferential recommendations for specific vaccines).

Primary updates and changes to the recommendations described in this report are of two types: 1) the vaccine virus composition for 2020–21 U.S. seasonal influenza vaccines and 2) recent regulatory actions, including two new influenza vaccine licensures that occurred after the publication of the 2019–20 ACIP Influenza Vaccine Statement (*18*). Information relevant to these changes includes the following:

1. Recommendations for the composition of Northern Hemisphere influenza vaccines are made by WHO, which organizes a consultation, generally in February of each year. Surveillance data are reviewed, and candidate vaccine viruses are discussed. A summary of the WHO meeting of February 28, 2020, for selection of the 2020–21 Northern Hemisphere vaccine viruses is available at https://www.who.int/influenza/vaccines/virus/recommendations/2020-21_north/en. Subsequently, FDA, which has regulatory authority over vaccines in the United States, convenes a meeting of its Vaccines and Related Biological Products Advisory Committee (VRBPAC). This committee considers the recommendations of WHO, reviews and discusses similar data, and makes a final decision regarding vaccine virus composition of influenza vaccines licensed and marketed in the United States. Summaries of the VRBPAC discussion of March 4, 2020, during which the composition of the 2020–21 U.S. influenza vaccines was discussed, are available at https://www.fda.gov/advisory-committees/advisory-committee-calendar/vaccines-and-related-biological-products-advisory-committee-march-4-2020-meeting-announcement.
2. Regarding recommendations concerning newly licensed influenza vaccines and changes to the licensed indications for existing vaccines, ACIP relies on FDA for review of safety, immunogenicity, and efficacy and effectiveness data pertaining to licensure of influenza vaccines. Regulatory information pertinent to the new vaccines discussed in this report is available at https://www.fda.gov/vaccines-blood-biologics/vaccines/fluzone-quadrivalent for Fluzone High-Dose Quadrivalent (HD-IIV4) and at https://www.fda.gov/vaccines-blood-biologics/fluad-quadrivalent for Fluad Quadrivalent (aIIV4).

## Primary Changes and Updates in the Recommendations

Routine annual influenza vaccination of all persons aged ≥6 months who do not have contraindications continues to be recommended. No preferential recommendation is made for one influenza vaccine product over another for persons for whom more than one licensed, recommended, and appropriate product is available. Updated information in this report includes the following:

1. The composition of the 2020–21 U.S. influenza vaccines includes updates to the influenza A(H1N1)pdm09, influenza A(H3N2), and influenza B/Victoria lineage components. These updated components will be included in both trivalent and quadrivalent vaccines. Quadrivalent vaccines will include an additional influenza B virus component from the B/Yamagata lineage, which is unchanged from that included in quadrivalent influenza vaccines used during the 2019–20 season. For the 2020–21 season, U.S. egg-based influenza vaccines (i.e., vaccines other than ccIIV4 and RIV4) will contain hemagglutinin (HA) derived from an influenza A/Guangdong-Maonan/SWL1536/2019 (H1N1)pdm09-like virus, an influenza A/Hong Kong/2671/2019 (H3N2)-like virus, an influenza B/Washington/02/2019 (Victoria lineage)-like virus, and (for quadrivalent egg-based vaccines) an influenza B/Phuket/3073/2013 (Yamagata lineage)-like virus. U.S. cell culture–based inactivated (ccIIV4) and recombinant (RIV4) influenza vaccines will contain HA derived from an influenza A/Hawaii/70/2019 (H1N1)pdm09-like virus, an influenza A/Hong Kong/45/2019 (H3N2)-like virus, an influenza B/Washington/02/2019 (Victoria lineage)-like virus, and an influenza B/Phuket/3073/2013 (Yamagata lineage)-like virus.
2. Two new influenza vaccine licensures are described:
  - In November 2019, FDA licensed Fluzone High-Dose Quadrivalent (HD-IIV4). Fluzone High-Dose Quadrivalent is approved for use in persons aged ≥65 years. For the 2020–21 season, Fluzone High-Dose Quadrivalent is expected to replace the previously available trivalent formulation of Fluzone High-Dose (HD-IIV3). The dose volume for Fluzone High-Dose Quadrivalent (0.7 mL) is slightly higher than that of trivalent Fluzone High-Dose (0.5 mL). Fluzone High-Dose Quadrivalent, like Fluzone High-Dose, contains 4 times the amount of HA per vaccine virus in each dose compared with standard-dose inactivated influenza vaccines (60 *μ*g per virus, versus 15 *μ*g in standard-dose IIVs).
  - In February 2020, FDA licensed Fluad Quadrivalent (aIIV4). Fluad Quadrivalent is approved for use in persons aged ≥65 years. For the 2020–21 season, both Fluad Quadrivalent and the previously licensed trivalent formulation of Fluad (aIIV3) are expected to be available. Fluad Quadrivalent, like Fluad, contains the adjuvant MF59.

Other changes include the following:

- Anatomic and functional asplenia; active communication between the cerebrospinal fluid (CSF) and oropharynx, nasopharynx, nose, or ear or any other cranial CSF leak; and cochlear implant have been added to the discussion of Contraindications and Precautions for the Use of LAIV4 and to Table 2.
- Discussion of Use of Influenza Antiviral Medications has been updated to reflect use of LAIV4 in the setting of use of newer influenza antiviral agents.
- Recommendations for Persons with a History of Egg Allergy now state that additional measures for those with a history of severe allergic reaction to egg (i.e., vaccination in a medical setting supervised by a health care provider who is able to recognize and manage severe allergic reactions) are needed only if a vaccine other than ccIIV4 or RIV4 is used.

## Recommendations for the Use of Influenza Vaccines, 2020–21

### Groups Recommended for Vaccination

Routine annual influenza vaccination is recommended for all persons aged ≥6 months who do not have contraindications. Recommendations regarding timing of vaccination, considerations for specific populations, the use of specific vaccines, and contraindications and precautions are summarized in the sections that follow.

### Timing of Vaccination

Balancing considerations regarding the unpredictability of timing of onset of the influenza season and concerns that vaccine-induced immunity might wane over the course of a season, vaccination is recommended to be offered by the end of October. Children aged 6 months through 8 years who require 2 doses (see Children Aged 6 Months Through 8 Years) should receive their first dose as soon as possible after the vaccine becomes available to allow the second dose (which must be administered ≥4 weeks later) to be received by the end of October. For those requiring only 1 dose for the season, early vaccination (i.e., in July and August) is likely to be associated with suboptimal immunity before the end of the influenza season, particularly among older adults. Community vaccination programs should balance maximizing the likelihood of persistence of vaccine-induced protection through the season with avoiding missed opportunities to vaccinate or vaccinating after onset of influenza circulation occurs. Efforts should be structured to optimize vaccination coverage before influenza activity in the community begins. Vaccination should continue to be offered as long as influenza viruses are circulating and unexpired vaccine is available. To avoid missed opportunities for vaccination, providers should offer vaccination during routine health care visits and hospitalizations. No recommendation is made for revaccination (i.e., providing a booster dose) later in the season of persons who have already been fully vaccinated.

The extent to which SARS-CoV-2, the novel coronavirus that causes COVID-19, will circulate during the 2020–21 influenza season is unknown. However, it is anticipated that SARS-CoV-2 and influenza viruses will both be active in the United States during the upcoming 2020–21 influenza season. Influenza vaccination programs might need to adapt and extend the duration of vaccination campaigns to accommodate stay-at-home orders and social distancing strategies aimed at slowing the spread of SARS-CoV-2. These circumstances might necessitate consideration of starting vaccination campaigns earlier (i.e., as soon as vaccine is available, which can be as early as July or August) to allow sufficient time to vaccinate the population and avoid some persons going unvaccinated for influenza. When possible, such considerations should be balanced against the potential waning of protection from influenza vaccination, particularly for persons aged ≥65 years. Additional information on SARS-CoV-2 illness is available on the CDC website (https://www.cdc.gov/coronavirus/2019-nCoV/index.html). Guidance for vaccine planning during the pandemic is available at https://www.cdc.gov/vaccines/pandemic-guidance/index.html. 

Optimally, vaccination should occur before onset of influenza activity in the community. However, because timing of the onset, peak, and decline of influenza activity varies, the ideal time to start vaccinating cannot be predicted each season. Moreover, more than one outbreak might occur in a given community in a single year. In the United States, localized outbreaks that indicate the start of seasonal influenza activity can occur as early as October. However, in 27 (75%) of 36 influenza seasons from 1982–83 through 2017–18, peak influenza activity (which often is close to the midpoint of influenza activity for the season) has not occurred until January or later, and in 21 (58%) seasons, the peak was in February or later (*20*). Activity peaked in February in 15 (42%) of these seasons (*20*).

Several observational studies (*21*–*29*) and a post hoc analysis from a randomized controlled trial (*30*) have reported decreases in vaccine effectiveness (VE) with increasing time postvaccination within a single influenza season. Waning effects have not been observed consistently across age groups, virus subtypes, and seasons, and observed decreases in protection could be at least in part attributable to bias, unmeasured confounding, or the late-season emergence of antigenic drift variants that are less well-matched to the vaccine viral strains. Some studies suggest waning occurs to a greater degree with influenza A(H3N2) viruses than with influenza A(H1N1) or influenza B viruses (*26*,*28*). This effect also might vary with recipient age; in some studies, waning was more pronounced among older adults (*21*,*23*) and younger children (*23*). Rates of decline in VE have also varied. A multiseason (2011–12 through 2014–15) analysis from the U.S. Influenza Vaccine Effectiveness (U.S. Flu VE) Network found that VE decreased by approximately 7% per month for influenza A(H3N2) and influenza B and 6%–11% per month for influenza A(H1N1)pdm09 (*25*). VE remained greater than zero for at least 5–6 months after vaccination. An analysis of the 2010–11 through 2013–14 seasons noted estimated effectiveness ranging from 54% to 67% during days 0–180 postvaccination; estimated VE was not statistically significant during the period between days 181 and 365 (*24*). A third multiseason analysis (2010–11 through 2014–15) conducted in Europe noted a decline in VE to 0% at 111 days postvaccination for influenza A(H3N2) viruses. VE against influenza B viruses decreased more slowly, and VE against influenza A(H1N1)pdm09 viruses remained roughly stable at 50%–55% through the influenza season (*28*).

Variable data concerning presence and rate of waning immunity after influenza vaccination, coupled with the unpredictable timing of the influenza season each year, prevent determination of an optimal time to vaccinate. Programmatic issues are also a consideration; although delaying vaccination might result in greater immunity later in the season, deferral also might result in missed opportunities to vaccinate as well as difficulties in vaccinating a population within a more constrained period. The potential contributions of these factors among persons aged ≥65 years have been assessed using a simulated mathematical model examining various scenarios of vaccination timing, timing of onset of the influenza season, rate of waning, and VE (*31*). In this model, assuming a historical average timing of onset of the influenza season, delaying vaccination until October resulted in more hospitalizations if >11% of persons aged ≥65 years who would have been vaccinated in August or September failed to get vaccinated. However, these predictions varied considerably with assumed timing of season onset, rate of waning immunity, and vaccine effectiveness.

Vaccination efforts should continue throughout the season because the duration of the influenza season varies, and influenza activity might not occur in certain communities until February or March. Providers should offer influenza vaccine routinely, and organized vaccination campaigns should continue throughout the influenza season, including after influenza activity has begun in the community. Although vaccination by the end of October is recommended, vaccine administered in December or later, even if influenza activity has already begun, might be beneficial in most influenza seasons. Providers should still offer influenza vaccination to unvaccinated persons who have already become ill with influenza during the season because the vaccine might protect them against other circulating influenza viruses.

### Guidance for Use in Specific Populations and Situations

#### Populations at Higher Risk for Medical Complications Attributable to Severe Influenza

All persons aged ≥6 months who do not have contraindications should be vaccinated annually. However, vaccination to prevent influenza is particularly important for persons who are at increased risk for severe illness and complications from influenza and for influenza-related outpatient, emergency department, or hospital visits. When vaccine supply is limited, vaccination efforts should focus on delivering vaccination to persons at higher risk for medical complications attributable to severe influenza who do not have contraindications. These persons include (no hierarchy is implied by order of listing):

- All children aged 6 through 59 months;
- All persons aged ≥50 years;
- Adults and children who have chronic pulmonary (including asthma), cardiovascular (excluding isolated hypertension), renal, hepatic, neurologic, hematologic, or metabolic disorders (including diabetes mellitus);
- Persons who are immunocompromised due to any cause (including but not limited to immunosuppression caused by medications or human immunodeficiency virus [HIV] infection);
- Women who are or will be pregnant during the influenza season;
- Children and adolescents (aged 6 months through 18 years) who are receiving aspirin- or salicylate-containing medications and who might be at risk for experiencing Reye syndrome after influenza virus infection;
- Residents of nursing homes and other long-term care facilities;
- American Indians/Alaska Natives; and
- Persons who are extremely obese (body mass index ≥40 for adults).

An IIV or RIV4 (as appropriate for the recipient’s age) is suitable for persons in all risk groups. LAIV4 is not recommended for some populations, including some of these listed groups. Contraindications and precautions to the use of LAIV4 are noted (Table 2).

#### Persons Who Live with or Care for Persons at Higher Risk for Influenza-Related Complications

All persons aged ≥6 months without contraindications should be vaccinated annually; however, in addition to persons at higher risk for medical complications attributable to severe influenza, emphasis also should be placed on vaccination of persons who live with or care for those who are at increased risk. When vaccine supply is limited, vaccination efforts should focus on delivering vaccination to persons at higher risk for influenza-related complications as well as persons who live with or care for such persons, including the following:

- Health care personnel, including all paid and unpaid persons working in health-care settings who have the potential for exposure to patients or to infectious materials. These personnel might include (but are not limited to) physicians, nurses, nursing assistants, nurse practitioners, physician assistants, therapists, technicians, emergency medical service personnel, dental personnel, pharmacists, laboratory personnel, autopsy personnel, students and trainees, contractual staff, and other persons not directly involved in patient care but who can potentially be exposed to infectious agents (e.g., clerical, dietary, housekeeping, laundry, security, maintenance, administrative, and billing staff and volunteers). ACIP guidance for vaccination of health care personnel has been published previously (*32*);
- Household contacts (including children aged ≥6 months) and caregivers of children aged ≤59 months (i.e., aged <5 years) and adults aged ≥50 years, particularly contacts of children aged <6 months; and
- Household contacts (including children aged ≥6 months) and caregivers of persons with medical conditions that put them at higher risk for severe complications from influenza.

Health care personnel and persons who are contacts of persons in these groups (with the exception of those of severely immunocompromised persons who require a protected environment) may receive any influenza vaccine that is otherwise indicated. Persons who care for severely immunocompromised persons requiring a protected environment should receive either IIV or RIV4. ACIP and the Healthcare Infection Control Practices Advisory Committee (HICPAC) have previously recommended that health care personnel who receive LAIV should avoid providing care for severely immunosuppressed patients requiring a protected environment for 7 days after vaccination and that hospital visitors who have received LAIV should avoid contact with such persons for 7 days after vaccination (*33*). However, such persons need not be restricted from caring for or visiting less severely immunosuppressed patients.

#### Influenza Vaccination of Persons with SARS-CoV-2 Infection (COVID-19)

Because SARS-CoV-2 is a novel coronavirus, clinical experience with influenza vaccination of persons with COVID-19 is limited. For those who have acute illness with suspected or laboratory-confirmed COVID-19, clinicians can consider delaying influenza vaccination until the patients are no longer acutely ill. If influenza vaccination is delayed, patients should be reminded to return for influenza vaccination once they have recovered from their acute illness.

#### Children Aged 6 Months through 8 Years

**Vaccines and dose volumes for children aged 6 through 35 months:** Children aged 6 through 35 months may receive any one of the four IIV4s licensed for this age group. The appropriate dose volumes for these vaccines differ for this age group (Table 3). For these vaccines, children aged 6 through 35 months may receive:

**TABLE 3:** Dose volumes for inactivated influenza vaccines licensed for children aged 6 through 35 months* — United States, 2020–21 influenza season

Trade name (Manufacturer)	Dose volume for children aged 6 through 35 mos (*μ*g HA per vaccine virus)
Afluria Quadrivalent (Seqirus)	0.25 mL (7.5 *μ*g)
Fluarix Quadrivalent (GlaxoSmithKline)	0.5 mL (15 *μ*g)
FluLaval Quadrivalent (GlaxoSmithKline)	0.5 mL (15 *μ*g)
Fluzone Quadrivalent (Sanofi Pasteur)	0.25 mL (7.5 *μ*g) or 0.5 mL (15 *μ*g)^†^

**Abbreviation:** HA = hemagglutinin.

* For persons aged ≥36 months (≥3 years), the dose volume is 0.5 mL for all inactivated influenza vaccines with the exception of Fluzone High-Dose Quadrivalent (HD-IIV4), which is licensed for persons aged ≥65 years and for which the volume is 0.7 mL per dose.

^†^ Fluzone Quadrivalent is currently licensed for this age group at either the 0.25-mL or 0.5-mL dose; however, 0.25-mL prefilled syringes are not expected to be available for 2020–21. If a prefilled syringe of Fluzone Quadrivalent is used for a child in this age group, the dose volume will be 0.5 mL per dose. Note that 0.5-mL vials should be accessed for only one dose, and multidose vials for only 10 doses, regardless of the volume of the doses taken or any remaining volume in the vial. Any vaccine remaining in a vial after the maximum number of doses has been removed should be discarded.

- 0.25 mL per dose of Afluria Quadrivalent (containing 7.5 *μ*g of HA per vaccine virus); or
- 0.5 mL per dose of Fluarix Quadrivalent (containing 15 *μ*g of HA per vaccine virus); or
- 0.5 mL per dose of FluLaval Quadrivalent (containing 15 *μ*g of HA per vaccine virus); or
- Either 0.25 mL per dose (containing 7.5 *μ*g of HA per vaccine virus) or 0.5 mL per dose (containing 15 *μ*g of HA per vaccine virus) of Fluzone Quadrivalent.

Alternatively, healthy children aged ≥2 years may receive LAIV4, 0.2 mL intranasally (0.1 mL in each nostril) (see Contraindications and Precautions for the Use of LAIV4; Table 2). LAIV4 is not licensed for children aged <2 years.

Care should be taken to administer an age-appropriate vaccine at the appropriate volume for each dose. For IIV4, the needed volume may be administered from a prefilled syringe containing the appropriate volume (as supplied by the manufacturer), a single-dose vial, or a multidose vial. Fluzone Quadrivalent is licensed for children aged 6 through 35 months at either 0.25 mL or 0.5 mL per dose. However, the 0.25-mL prefilled syringes are not anticipated to be available for the 2020–21 season. If a prefilled syringe of Fluzone Quadrivalent is used for a child in this age group, the dose volume will be 0.5 mL per dose. Single-dose, 0.5-mL vials should be accessed for only 1 dose, and multidose vials for only 10 doses, regardless of the volume of the doses taken or any remaining volume in the vial. Any vaccine remaining in a vial after the maximum number of doses has been removed should be discarded.

**Number of doses for children aged 6 months through 8 years:** Children aged 6 months through 8 years require 2 doses of influenza vaccine administered a minimum of 4 weeks apart during their first season of vaccination for optimal protection (*34*–*37*). Determination of the number of doses needed is based on 1) the child’s age at the time of the first dose of 2020–21 influenza vaccine and 2) the number of doses of influenza vaccine received in previous influenza seasons:

- For those aged 6 months through 8 years, the number of doses of influenza vaccine needed for the 2020–21 influenza season is determined as follows (Figure):

**FIGURE:**
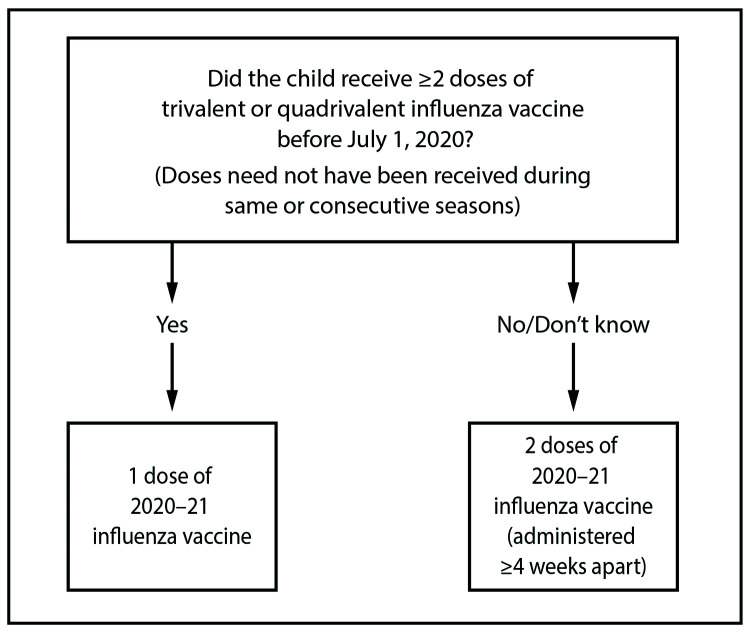
Influenza vaccine dosing algorithm for children aged 6 months through 8 years* — Advisory Committee on Immunization Practices, United States, 2020–21 influenza season * For children aged 8 years who require 2 doses of vaccine, both doses should be administered even if the child turns age 9 years between receipt of dose 1 and dose 2.
  - Those who have previously received ≥2 total doses of trivalent or quadrivalent influenza vaccine ≥4 weeks apart before July 1, 2020, require only 1 dose for the 2020–21 season. The 2 previous doses of influenza vaccine do not need to have been administered in the same season or consecutive seasons.
  - Those who have not previously received ≥2 doses of trivalent or quadrivalent influenza vaccine ≥4 weeks apart before July 1, 2020, or whose previous influenza vaccination history is unknown, require 2 doses for the 2020–21 season. The interval between the 2 doses should be ≥4 weeks. Two doses are recommended even if the child turns age 9 years between receipt of dose 1 and dose 2.
- Adults and children aged ≥9 years need only 1 dose of influenza vaccine for the 2020–21 season.

#### Pregnant Women

Pregnant and postpartum women have been observed to be at higher risk for severe illness and complications from influenza, particularly during the second and third trimesters. ACIP and the American College of Obstetricians and Gynecologists recommend that all women who are pregnant or who might be pregnant or postpartum during the influenza season receive influenza vaccine (*38*,*39*). Any licensed, recommended, and age-appropriate IIV or RIV4 may be used. LAIV4 should not be used during pregnancy. Influenza vaccine can be administered at any time during pregnancy, before and during the influenza season.

Although experience with the use of IIVs during pregnancy is substantial, data specifically reflecting administration of influenza vaccines during the first trimester are relatively limited (see Safety of Influenza Vaccines: Pregnant Women and Neonates in the supplementary Background Document). Most studies have not noted an association between influenza vaccination and adverse pregnancy outcomes, including spontaneous abortion (*40*–*50*). One observational Vaccine Safety Datalink (VSD) study conducted during the 2010–11 and 2011–12 seasons noted an association between receipt of IIV containing influenza A(H1N1)pdm09 and risk for spontaneous abortion (miscarriage) in the 28 days after IIV, when an H1N1pdm09-containing vaccine had also been received the previous season (*51*). However, in a larger VSD follow-up study, IIV was not associated with an increased risk for spontaneous abortion during the 2012–13, 2013–14, and 2014–15 seasons, regardless of previous season vaccination (*52*).

Substantially less experience exists with more recently licensed IIV products (e.g., quadrivalent and cell culture–based vaccines) during pregnancy as compared with previously available products. For RIV (available as RIV3 from 2013–14 through 2017–18 and as RIV4 since 2017–18), data are limited to reports of pregnancies occurring incidentally during clinical trials, Vaccine Adverse Event Reporting System (VAERS) reports, and pregnancy registries. Pregnancy registries and surveillance studies exist for some products; information can be found in package inserts at https://www.fda.gov/vaccines-blood-biologics/vaccines/influenza-virus-vaccine-trivalent-types-and-b for trivalent vaccines and at https://www.fda.gov/vaccines-blood-biologics/approved-products/influenza-virus-vaccine-quadrivalent-types-and-types-b for quadrivalent vaccines.

#### Older Adults

Because of the vulnerability of older adults to severe influenza illness, hospitalization, and death, efficacy and effectiveness of influenza vaccines in this population is an area of active research (see Immunogenicity, Efficacy, and Effectiveness of Influenza Vaccines: HD-IIV3, aIIV3, and RIV4 for Older Adults in the Background Document). Recent comparative studies of vaccine efficacy and effectiveness against laboratory-confirmed influenza outcomes among older adults have focused on Fluzone High-Dose (HD-IIV3) (*53*–*55*), Flublok Quadrivalent (RIV4) (*56*), and Fluad (aIIV3) (*57*) (see Table in Background Document). These studies have evaluated each of these three vaccines compared with standard-dose, nonadjuvanted IIV (SD-IIV3). To date, HD-IIV3 has been the most extensively studied in this regard, and evidence has accumulated for its superior efficacy and effectiveness compared with SD-IIV3 in this population. For the 2020–21 season, newly licensed quadrivalent formulations of high-dose (HD-IIV4) and adjuvanted (aIIV4) influenza vaccines are expected to be available. Data summarizing comparisons of these newer quadrivalent formulations relative to standard-dose nonadjuvanted IIV4 against laboratory-confirmed influenza outcomes are not yet available. Moreover, data from studies comparing the efficacy or effectiveness of HD-IIVs, aIIVs, and RIV4 directly with one another against laboratory-confirmed influenza outcomes among older adults are limited, which prevents recommending one of these three vaccines over another for this population. In comparative safety studies, some injection site and systemic reactions were observed more frequently in older persons vaccinated with HD-IIV3 and aIIV3 compared with nonadjuvanted SD-IIV3 (*58*,*59*).

Fluzone High-Dose (HD-IIV3) met prespecified criteria for superior efficacy against laboratory-confirmed influenza compared with that of standard-dose Fluzone (SD-IIV3) in a randomized trial conducted over two seasons (2011–12 and 2012–13) among 31,989 persons aged ≥65 years (*53*,*60*,*61*). For the primary outcome (prevention of laboratory-confirmed influenza caused by any viral type or subtype and associated with protocol-defined influenza-like illness [ILI]), the relative efficacy of Fluzone HD-IIV3 compared with Fluzone SD-IIV3 was 24.2% (95% confidence interval [CI]: 9.7%–36.5%). These findings are further supported by results from retrospective studies of Centers for Medicare and Medicaid Services and Veterans Administration data, as well as a cluster-randomized trial of HD-IIV3 compared with SD-IIV among older adults in nursing homes (*62*–*66*). A meta-analysis reported that HD-IIV3 provided better protection than SD-IIV3 against ILI (relative VE = 19.5%; 95% CI: 8.6%–29.0%); all-cause hospitalizations (relative VE = 9.1%; 95% CI: 2.4%–15.3%); and hospitalizations due to influenza (relative VE = 17.8%; 95% CI: 8.1%–26.5%), pneumonia (relative VE = 24.3%; 95% CI: 13.9%–33.4%), and cardiorespiratory events (relative VE = 18.2%; 95% CI: 6.8%–28.1%) (*67*). For the 2020–21 season, HD-IIV3 is expected to be replaced by Fluzone High-Dose Quadrivalent (HD-IIV4). HD-IIV4 exhibited noninferior immunogenicity to HD-IIV3 in a two-season randomized trial (*68*); estimates of relative efficacy or effectiveness compared with standard-dose nonadjuvanted IIV4 are not available.

In an exploratory analysis of data from a single-season (2014–15) randomized trial conducted among 8,604 adults aged ≥50 years, Flublok Quadrivalent (RIV4) was more efficacious than SD-IIV4 (*56*,*69*); however, no claim of superiority was approved for the package insert (*69*). For the primary outcome (protocol-defined ILI, caused by any influenza virus type or subtype, and confirmed by reverse transcription–polymerase chain reaction), the relative VE of RIV4 compared with SD-IIV4 was 30% (95% CI: 10%–47%). When restricted to persons aged ≥65 years, the relative VE of RIV4 was 17% (95% CI: −20% to 43%).

No data from randomized controlled trials are available concerning efficacy of Fluad (aIIV3) compared with nonadjuvanted IIV3 against laboratory-confirmed influenza outcomes in older adults. In an observational study from a single season (2011–12), aIIV3 was more effective against laboratory-confirmed influenza than nonadjuvanted SD-IIV3 among adults aged ≥65 years (N = 227, 165 of whom received aIIV3 and 62 SD-IIV3) (*57*). The relative effectiveness of aIIV3 compared with nonadjuvanted SD-IIV3 was 63% (95% CI: 4%–86%). aIIV3 was associated with reduced risk for hospitalization for pneumonia and influenza diagnoses (*70*) and pneumonia, cerebrovascular, or cardiovascular diagnoses relative to nonadjuvanted IIV3 in studies of medical record data (*71*). For the 2020–21 season, Fluad Quadrivalent (aIIV4) is expected to be available. This new quadrivalent formulation met prespecified immunogenicity criteria relative to a noninfluenza control vaccine in a randomized trial (*72*). Data comparing the efficacy or effectiveness of aIIV4 with that of nonadjuvanted IIV4 against laboratory-confirmed influenza outcomes are not yet available.

ACIP will continue to review data on the efficacy and effectiveness of these vaccines as more information emerges. No preference is expressed for any one vaccine type. Vaccination should not be delayed if a specific product is not readily available. For persons aged ≥65 years, any age-appropriate IIV formulation (standard dose or high dose, trivalent or quadrivalent, nonadjuvanted or adjuvanted) or RIV4 is an acceptable option.

#### Immunocompromised Persons

ACIP recommends that persons with immunocompromising conditions (including but not limited to persons with congenital and acquired immunodeficiency states, persons who are immunocompromised due to medications, and persons with anatomic and functional asplenia) should receive an age-appropriate IIV or RIV4. ACIP recommends that LAIV4 not be used for these groups because of the uncertain but biologically plausible risk for disease attributable to the live vaccine virus. Use of LAIV4 in persons with these and other conditions is discussed in more detail (see Dosage, Administration, Contraindications, and Precautions) (Table 2).

Immunocompromised states comprise a heterogeneous range of conditions with varying risks for severe infections. In many instances, limited data are available regarding the use of influenza vaccines in the setting of specific immunocompromised states. Timing of vaccination might be a consideration (e.g., vaccinating during some period either before or after an immunocompromising intervention). The Infectious Diseases Society of America (IDSA) has published detailed guidance for the selection and timing of vaccines for persons with specific immunocompromising conditions (see Additional Resources). Immune response to influenza vaccines might be blunted in persons with some conditions, such as persons with congenital immune deficiencies, and persons receiving cancer chemotherapy or immunosuppressive medications.

#### Persons with a History of Guillain-Barré Syndrome After Influenza Vaccination

A history of Guillain-Barré syndrome (GBS) within 6 weeks of a previous dose of any type of influenza vaccine is considered a precaution to vaccination (Table 2). Persons who are not at higher risk for severe influenza complications (see Populations at Higher Risk for Medical Complications Attributable to Severe Influenza) and who are known to have experienced GBS within 6 weeks of a previous influenza vaccination generally should not be vaccinated. As an alternative to vaccination, providers might consider using influenza antiviral chemoprophylaxis for these persons (*73*). However, the benefits of influenza vaccination might outweigh the possible risks for certain persons who have a history of GBS within 6 weeks after receipt of influenza vaccine and who also are at higher risk for severe complications from influenza.

#### Persons with a History of Egg Allergy

As is the case for all vaccines, influenza vaccines contain various components that might cause allergic and anaphylactic reactions. Not all such reactions are related to egg proteins; however, the possibility of reactions to influenza vaccines in egg-allergic persons might be of concern to these persons and vaccine providers. Currently available influenza vaccines, with the exceptions of RIV4 (Flublok Quadrivalent, licensed for those aged ≥18 years) and ccIIV4 (Flucelvax Quadrivalent, licensed for those aged ≥4 years), are prepared by propagation of virus in embryonated eggs and might contain trace amounts of egg proteins, such as ovalbumin.

Severe allergic reactions to vaccines, although rare, can occur at any time, even in the absence of a history of previous allergic reaction. Therefore, all vaccine providers should be familiar with the office emergency plan and be certified in cardiopulmonary resuscitation (*74*). For persons who report a history of egg allergy, ACIP recommends the following:

- Persons with a history of egg allergy who have experienced only urticaria (hives) after exposure to egg should receive influenza vaccine. Any licensed, recommended influenza vaccine (i.e., any IIV, RIV4, or LAIV4) that is otherwise appropriate for the recipient’s age and health status may be used.
- Persons who report having had reactions to egg involving symptoms other than urticaria (e.g., angioedema or swelling, respiratory distress, lightheadedness, or recurrent vomiting) or who required epinephrine or another emergency medical intervention may similarly receive any licensed, recommended influenza vaccine (i.e., any IIV, RIV4, or LAIV4) that is otherwise appropriate for their age and health status. If a vaccine other than ccIIV4 or RIV4 is used, the selected vaccine should be administered in an inpatient or outpatient medical setting (including but not necessarily limited to hospitals, clinics, health departments, and physician offices). Vaccine administration should be supervised by a health care provider who is able to recognize and manage severe allergic reactions.
- A previous severe allergic reaction to influenza vaccine, regardless of the component suspected of being responsible for the reaction, is a contraindication to future receipt of the vaccine.

No postvaccination observation period is recommended specifically for egg-allergic persons. However, ACIP recommends that vaccine providers consider observing patients (seated or supine) for 15 minutes after administration of any vaccine to decrease the risk for injury should syncope occur (*74*).

#### Vaccination Issues for Travelers

In temperate climate regions of the Northern and Southern hemispheres, influenza activity is seasonal, occurring approximately from October–May in the Northern Hemisphere and April–September in the Southern Hemisphere. In the tropics, influenza might occur throughout the year. Travelers can be exposed to influenza when traveling to an area where influenza is circulating or when traveling as part of large tourist groups (e.g., on cruise ships) that include persons from areas of the world where influenza viruses are circulating (*75*–*78*).

Travelers who want to reduce the risk for influenza should consider influenza vaccination, preferably at least 2 weeks before departure. In particular, persons who live in the United States and are at higher risk for complications of influenza and who were not vaccinated with influenza vaccine during the previous Northern Hemisphere fall or winter should consider receiving influenza vaccine before departure if they plan to travel to the tropics, to the Southern Hemisphere during the Southern Hemisphere influenza season (April–September), or with organized tourist groups or on cruise ships to any location. Persons at higher risk who received the previous season’s vaccine before travel should consult with their health care provider to discuss the risk for influenza or other travel-related diseases before embarking on travel during the summer. All persons (regardless of risk status) vaccinated before travel should receive the current vaccine the following fall or winter.

Influenza vaccine formulated for the Southern Hemisphere might differ in viral composition from the Northern Hemisphere vaccine. For persons traveling to the Southern Hemisphere during the Southern Hemisphere influenza season, receipt of a current U.S.-licensed Southern Hemisphere formulation influenza vaccine before departure might be reasonable but might not be feasible because of limited access to or unavailability of this vaccine in the United States. With the exception of the Southern Hemisphere formulation of Fluzone Quadrivalent (IIV4), Southern Hemisphere formulation seasonal influenza vaccines are not licensed in the United States and generally are not commercially available in the United States. More information on influenza vaccines and travel is available at https://wwwnc.cdc.gov/travel/diseases/influenza-seasonal-zoonotic-and-pandemic.

#### Use of Influenza Antiviral Medications

Administration of IIV or RIV4 to persons receiving influenza antiviral medications for treatment or chemoprophylaxis is acceptable. Data concerning vaccination with LAIV4 in the setting of influenza antiviral use are not available. However, influenza antiviral medications might interfere with the action of LAIV4 because it contains live virus.

The package insert for LAIV4 notes that antiviral agents might reduce the effectiveness of the vaccine if given within the interval from 48 hours before to 14 days after vaccination (*79*). However, the newer influenza antivirals peramivir and baloxavir have longer half-lives than oseltamivir and zanamivir (approximately 20 hours for peramivir [*80*] and 79 hours for baloxavir [*81*]) and could conceivably interfere with the replication of LAIV4 if administered >48 hours before vaccination. Potential interactions between influenza antivirals and LAIV4 have not been studied, and the ideal intervals between administration of these medications and LAIV4 is not known. Assuming a period of at least 5 half-lives for substantial decline in drug levels (*82*), it is reasonable to assume that peramivir might interfere with the mechanism of LAIV4 if given from 5 days before through 2 weeks after vaccination, and baloxavir might interfere if given from 17 days before through 2 weeks after vaccination. The interval between influenza antiviral receipt and LAIV4 for which interference might potentially occur might be further prolonged in the presence of medical conditions that delay medication clearance (e.g., renal insufficiency). Persons who receive these medications during these periods before or after receipt of LAIV4 should be revaccinated with another appropriate influenza vaccine (e.g., IIV or RIV4). 

#### Administration of Influenza Vaccines with Other Vaccines

IIVs and RIV4 may be administered concomitantly or sequentially with other inactivated vaccines or live vaccines. Injectable vaccines that are given concomitantly should be administered at separate anatomic sites. LAIV4 may be administered simultaneously with other live or inactivated vaccines. However, if two live vaccines are not given simultaneously, then after administration of one live vaccine (such as LAIV4), at least 4 weeks should pass before another live vaccine is administered (*74*).

Relatively limited data are available on the concomitant administration of influenza vaccines with other vaccines. Studies of live attenuated zoster vaccine and IIV3 (*83*) or IIV4 (*84*) among persons aged ≥50 years noted similar antibody responses whether the two vaccines were administered concomitantly or 4 weeks apart. In some studies, reduced responses have been noted to 13-valent pneumococcal conjugate vaccine (PCV13) (*85*,*86*), tetanus antigens (*87*), and pertussis antigens (*87*) when co-administered with IIV3 to adults; in most instances, the clinical significance of this is uncertain. Simultaneous administration of IIV4 and 23-valent pneumococcal polysaccharide vaccine (PPSV23) to persons aged ≥65 years was associated with lower seroprotection rates to one influenza B antigen at 4–6 weeks postvaccination as compared with sequential administration 2 weeks apart; seroprotection was not significantly different between the two groups for any of the four influenza antigens at 6 months postvaccination (*88*). Reassuring safety profiles have been noted for simultaneous administration of IIVs with live attenuated zoster vaccine (*83*,*84*), PCV13 (*85*,*86*), PPSV23 (*88*,*89*), and tetanus toxoid, reduced diphtheria toxoid, and acellular pertussis (Tdap) vaccine among adults (*87*) and of Tdap among pregnant women (*90*). Although increased prevalence of injection site or systemic adverse reactions has been noted with concurrent administration in some of these studies, these symptoms have generally been reported to be mild or moderate.

Among children aged 6 through 23 months, co-administration of IIV and PCV13 was associated with increased risk for fever on the day of vaccination and the day after (i.e., days 0–1 postvaccination) in an observational study conducted during the 2011–12 season (*91*). A randomized clinical trial during the 2017–18 influenza season suggested that delaying IIV4 administration by 2 weeks in children receiving DTaP and PCV13 did not reduce fever prevalence after vaccination (*92*). Increased risk for febrile seizures in this age group has been noted within days 0–1 after co-administration of IIV with PCV7, PCV13, or diphtheria and tetanus toxoids and acellular pertussis (DTaP) vaccines during the 2006–07 through 2010–11 seasons (*93*) and with PCV13 during the 2014–15 season (*94*). Although of concern to parents, most febrile seizures are brief and have a good prognosis (*95*). After considering the risks and benefits, no changes in the recommendations for administration of these vaccines were made, and these vaccines can be given concomitantly. Surveillance of febrile seizures is ongoing through VAERS, and the VSD annual influenza safety surveillance includes monitoring for seizures after vaccinations.

Studies of concomitant administration of LAIV with other vaccines are limited. Concurrent administration of LAIV3 with measles, mumps, and rubella (MMR) and varicella vaccine to children was not associated with diminished immunogenicity to antigens in any of the vaccines in one study (*96*); diminished response to rubella was observed in another study examining co-administration of LAIV3 and MMR (*97*). No safety concerns were noted in these studies.

In recent years, several vaccines containing nonaluminum adjuvants have been licensed for use in the United States for the prevention of various infectious diseases. These include AS01_B_ (in Shingrix, recombinant zoster subunit vaccine) (*98*); MF59 (in Fluad [aIIV3] and Fluad Quadrivalent [aIIV4]) (*72*,*99*); and cytosine phosphoguanine oligodeoxynucleotide (in Heplisav-B, recombinant hepatitis B surface antigen vaccine) (*100*). Data are limited regarding co-administration of these vaccines with other adjuvanted or nonadjuvanted vaccines. Co-administration of Shingrix with nonadjuvanted IIV4 has been studied; no evidence of decreased immunogenicity or safety concerns was noted (*101*). The immunogenicity and safety of simultaneous or sequential administration of two nonaluminum-adjuvant–containing vaccines has not been evaluated, and the ideal interval between such vaccines when given sequentially is not known. In the study of Shingrix and IIV4 (*101*), most reactogenicity symptoms resolved within 4 days. Because of the limited data on the safety of simultaneous administration of two or more vaccines containing nonaluminum adjuvants and the availability of nonadjuvanted influenza vaccine options, selection of a nonadjuvanted influenza vaccine may be considered in situations in which influenza vaccine and another vaccine containing a nonaluminum adjuvant are to be administered concomitantly. However, vaccination should not be delayed if a specific product is not available. As recommended for all vaccines, vaccines with nonaluminum adjuvants should be administered at separate anatomic sites from other vaccines that are given concomitantly (*74*).

## Influenza Vaccine Composition and Available Vaccines

### Influenza Vaccine Composition for the 2020–21 Season

All influenza vaccines licensed in the United States will contain components derived from influenza viruses antigenically similar to those recommended by FDA (https://www.fda.gov/advisory-committees/advisory-committee-calendar/vaccines-and-related-biological-products-advisory-committee-march-4-2020-meeting-announcement). Most influenza vaccines available in the United States for the 2020–21 season will be quadrivalent vaccines, with the exception of MF59-adjuvanted IIV (aIIV), which is expected to be available in both trivalent (aIIV3, Fluad) and quadrivalent (aIIV4, Fluad Quadrivalent) formulations.

For the 2020–21 season, U.S. egg-based influenza vaccines (i.e., vaccines other than ccIIV4 and RIV4) will contain HA derived from

- an influenza A/Guangdong-Maonan/SWL1536/2019 (H1N1)pdm09-like virus;
- an influenza A/Hong Kong/2671/2019 (H3N2)-like virus;
- an influenza B/Washington/02/2019 (Victoria lineage)-like virus; and
- for quadrivalent vaccines only, an influenza B/Phuket/3073/2013 (Yamagata lineage)-like virus.

For the 2020–21 season, U.S. cell culture–based inactivated (ccIIV4) and recombinant (RIV4) influenza vaccines will contain HA derived from

- an influenza A/Hawaii/70/2019 (H1N1)pdm09-like virus;
- an influenza A/Hong Kong/45/2019 (H3N2)-like virus;
- an influenza B/Washington/02/2019 (Victoria lineage)-like virus; and
- an influenza B/Phuket/3073/2013 (Yamagata lineage)-like virus.

The 2020–21 composition reflects updates in the influenza A(H1N1)pdm09, influenza A(H3N2), and influenza B (Victoria lineage) components.

### Vaccines Available for the 2020–21 Season

Various influenza vaccines will be available for the 2020–21 season (Table 1). For many vaccine recipients, more than one type or brand of vaccine might be appropriate within approved indications and ACIP recommendations. A licensed influenza vaccine that is appropriate for the recipient’s age and health status should be used. Specific age indications for licensed influenza vaccines are summarized (Table 1); current prescribing information should be consulted for authoritative, up-to-date information. Contraindications and precautions for the different types of influenza vaccines are summarized (Table 2).

Not all influenza vaccines are likely to be uniformly available in any given practice setting or geographic locality. Vaccination should not be delayed to obtain a specific product when an appropriate one is already available. Within these guidelines and approved indications, when more than one type of vaccine is available and appropriate, no preferential recommendation is made for the use of any one influenza vaccine over another.

Since the publication of the previous season’s guidance, two new influenza vaccines have been licensed. These include the licensure of Fluzone High-Dose Quadrivalent in November 2019 and of Fluad Quadrivalent in February 2020. Both of these influenza vaccines are expected to be available in the United States during the 2020–21 season. New licensures and changes to FDA-approved labeling might occur after publication of this report. As these changes occur and new vaccines become available, they will be reflected in the online version of Table 1, available at https://www.cdc.gov/flu/professionals/acip/2020-2021/acip-table.htm.

### Dosage, Administration, Contraindications, and Precautions

#### Inactivated Influenza Vaccines (IIVs)

**Available vaccines:** As in recent seasons, various inactivated influenza vaccines (IIVs) are expected to be available for 2020–21 (Table 1). Certain IIVs are licensed for persons as young as age 6 months. However, licensed age indications differ for different products. Moreover, for some IIVs, the dose volume for children aged 6 through 35 months differs from that for older children and adults (Table 3). Care should be taken to administer the appropriate dose volume of an age-appropriate product to each recipient.

Standard-dose, nonadjuvanted IIVs contain 15 *μ*g of HA per vaccine virus in a 0.5-mL dose (7.5 *μ*g of HA per vaccine virus in a 0.25-mL dose). For 2020–21, this category is expected to include five different products, all of which will be quadrivalent (IIV4s) (Table 1). Four of these vaccines are egg based, and one is cell culture based. Egg-based and cell culture–based vaccines differ in the substrate in which reference vaccine viruses supplied to the manufacturer are propagated in quantities sufficient to produce the needed number of doses of vaccine. The egg-based IIV4s, Afluria Quadrivalent (*102*), Fluarix Quadrivalent (*103*), FluLaval Quadrivalent (*104*), and Fluzone Quadrivalent (*105*), are licensed for persons aged ≥6 months. The cell culture–based IIV4, Flucelvax Quadrivalent (ccIIV4), is licensed for persons aged ≥4 years. For the manufacture of ccIIV4, the influenza vaccine viruses are propagated in Madin-Darby canine kidney cells instead of eggs (*106*).

Three additional IIVs that will be available for the 2020–21 season are licensed for persons aged ≥65 years. These vaccines are egg based. Fluzone High-Dose Quadrivalent contains 60 *μ*g of HA per vaccine virus (240 *μ*g total) in a 0.7-mL dose (*68*). For 2020–21, this quadrivalent formulation of the high-dose IIV is expected to replace the previous trivalent formulation. Adjuvanted inactivated influenza vaccine (aIIV), which contains MF59 adjuvant, is expected to be available in both trivalent (Fluad, aIIV3) and quadrivalent (Fluad Quadrivalent, aIIV4) formulations for 2020–21. Both contain 15 *μ*g of HA per vaccine virus in each 0.5-mL dose (45 *μ*g total for Fluad and 60 *μ*g total for Fluad Quadrivalent) (*72*,*99*).

**Dosage and administration:** For children aged 6 through 35 months, four IIV4s are expected to be available for the 2020–21 season: Afluria Quadrivalent, Fluarix Quadrivalent, FluLaval Quadrivalent, and Fluzone Quadrivalent. The approved dose volumes for these vaccines differ for this age group (Table 3). For each of these IIV4s, a 0.5-mL dose contains 15 *μ*g of HA per vaccine virus, whereas a 0.25-mL dose contains 7.5 *μ*g of HA per vaccine virus. Care should be taken to administer the appropriate dose volume for the particular product. If prefilled syringes are not available, the appropriate volume may be administered from a single-dose or multidose vial. If a 0.5-mL single-dose vial is used for a 0.25-mL dose for a child aged 6 through 35 months, only half the vial volume should be administered, and the remaining half should be discarded. Of note, dose volume is distinct from the number of doses. Children in this age group who require 2 doses for 2020–21 (see Children Aged 6 Months through 8 Years; Figure) need 2 separate doses administered ≥4 weeks apart, regardless of the specific IIV4 used and volume given for each dose.

For children aged 36 months (3 years) through 17 years and adults aged ≥18 years, the dose volume for IIVs is 0.5 mL per dose, with the exception of Fluzone High-Dose Quadrivalent (HD-IIV4, licensed for persons aged ≥65 years), for which the correct volume is 0.7 mL per dose. If a smaller vaccine dose (e.g., 0.25 mL) is inadvertently administered to a person aged ≥36 months, the remaining volume needed to make a full dose should be administered during the same vaccination visit. If the error is discovered later (after the recipient has left the vaccination setting), a full dose should be administered as soon as the recipient can return (i.e., 0.7 mL for HD-IIV4 and 0.5 mL for all other IIVs). Vaccination with a formulation approved for adult use should be counted as a single dose if inadvertently administered to a child.

IIVs are administered intramuscularly (IM). For adults and older children, the deltoid is the preferred site. Infants and younger children should be vaccinated in the anterolateral thigh. Additional specific guidance regarding site selection and needle length for IM injection is provided in the ACIP General Best Practice Guidelines for Immunization (*74*).

One IIV4, Afluria Quadrivalent, is licensed for IM injection via the PharmaJet Stratis jet injector for persons aged 18 through 64 years (*102*). Persons in this age group may receive Afluria Quadrivalent via either needle and syringe or this specific jet injection device. Children aged 6 months through 17 years and adults aged ≥65 years should receive this vaccine by needle and syringe only. No other IIVs are licensed for administration by jet injector.

**Trivalent versus quadrivalent IIVs:** For the 2020–21 influenza season, all IIVs will be quadrivalent, with the exception of adjuvanted IIV (aIIV), which is expected to be available in both trivalent (aIIV3) and quadrivalent (aIIV4) formulations. Quadrivalent and trivalent vaccines are alike in that they contain two influenza A viruses (an A[H1N1] virus and an A[H3N2] virus). They differ with regard to the number of influenza B viruses. Quadrivalent vaccines (IIV4s) contain one virus from each of the two influenza B virus lineages (Yamagata and Victoria), whereas trivalent vaccines (IIV3s) contain one influenza B virus from one lineage (for 2020–21, a Victoria lineage virus). IIV4s are thus designed to provide broader protection against circulating influenza B virus strains. However, no preference is expressed for either quadrivalent or trivalent vaccines.

**Contraindications and precautions for the use of IIVs:** Manufacturer package inserts and updated CDC and ACIP guidance should be consulted for information on contraindications and precautions for individual influenza vaccines. A history of a severe allergic reaction to the vaccine or any of its components (which include egg for all IIVs except for Flucelvax Quadrivalent [ccIIV4]) is a labeled contraindication to the receipt of IIVs (Table 2). However, ACIP makes specific recommendations for the use of influenza vaccine for persons with egg allergy (see Persons with a History of Egg Allergy). Influenza vaccine is not recommended for persons with a history of severe allergic reaction to the vaccine or to components other than egg. Information about vaccine components can be found in the package inserts for each vaccine. Prophylactic use of antiviral agents is an option that can be considered for preventing influenza among persons who cannot receive vaccine, particularly for those who are at higher risk for medical complications attributable to severe influenza (*73*).

Moderate or severe acute illness with or without fever is a general precaution for vaccination (*74*). A history of GBS within 6 weeks after receipt of a previous dose of influenza vaccine is considered a precaution for the use of all influenza vaccines (Table 2).

#### Recombinant Influenza Vaccine (RIV4)

**Available vaccines:** One recombinant influenza vaccine, Flublok Quadrivalent (RIV4), is expected to be available during the 2020–21 influenza season. RIV4 is indicated for persons aged ≥18 years. This vaccine contains recombinant HA produced in an insect cell line using genetic sequences from cell-derived influenza viruses and is manufactured without the use of influenza viruses or eggs (*69*). No preference is expressed for RIV4 versus other influenza vaccines used within specified indications.

**Dosage and administration:** RIV4 is administered by IM injection via needle and syringe. A 0.5-mL dose contains 45 *μ*g of HA derived from each vaccine virus (180 *μ*g total).

**Contraindications and precautions for the use of RIV4:** RIV4 is contraindicated in persons who have had a severe allergic reaction to any component of the vaccine. Moderate or severe acute illness with or without fever is a general precaution for vaccination (*74*). A history of GBS within 6 weeks after receipt of a previous dose of influenza vaccine is considered a precaution for the use of all influenza vaccines (Table 2). RIV4 is not licensed for children aged <18 years.

#### Live Attenuated Influenza Vaccine (LAIV4)

**Available vaccines***:* One live attenuated influenza vaccine, FluMist Quadrivalent (LAIV4), is expected to be available during the 2020–21 influenza season. LAIV4 is licensed for persons aged 2 through 49 years.

**Dosage and administration:** LAIV4 is administered intranasally using the supplied prefilled, single-use sprayer containing 0.2 mL of vaccine. Approximately 0.1 mL (i.e., half the total sprayer contents) is sprayed into the first nostril while the recipient is in the upright position. An attached dose-divider clip is removed from the sprayer to permit administration of the second half of the dose into the other nostril. If the recipient sneezes immediately after administration, the dose should not be repeated. However, if nasal congestion is present that might impede delivery of the vaccine to the nasopharyngeal mucosa, deferral of administration should be considered until resolution of the illness, or another appropriate vaccine should be administered instead.

**Contraindications and precautions for the use of LAIV4:** Conditions considered by ACIP to be contraindications and precautions to the use of LAIV4 are summarized (Table 2). These include two labeled contraindications that appear in the package insert (*79*) and other conditions for which there is uncertain but biologically plausible potential risk associated with live viruses or limited data for use of LAIV.

Contraindications to use of LAIV4 include the following:

- Severe allergic reaction to any component of the vaccine or to a previous dose of any influenza vaccine (a labeled contraindication noted in the package insert). However, ACIP makes an exception for allergy to egg (see Persons with a History of Egg Allergy)
- Children and adolescents receiving concomitant aspirin- or salicylate-containing medications (Table 2), because of the potential risk for Reye syndrome (a labeled contraindication noted in the package insert)
- Children aged 2 through 4 years who have received a diagnosis of asthma or whose parents or caregivers report that a health care provider has told them during the past 12 months that their child had wheezing or asthma or whose medical record indicates a wheezing episode has occurred during the preceding 12 months
- Children and adults who are immunocompromised due to any cause, including but not limited to immunosuppression caused by medications, congenital or acquired immunodeficiency states, HIV infection, anatomic asplenia, or functional asplenia (such as that due to sickle cell anemia)
- Close contacts and caregivers of severely immunosuppressed persons who require a protected environment
- Pregnancy
- Persons with active communication between the cerebrospinal fluid (CSF) and the oropharynx, nasopharynx, nose, or ear or any other cranial CSF leak
- Persons with cochlear implants, because of the potential for CSF leak, which might exist for some period after implantation (providers might consider consulting with a specialist concerning the risk for persistent CSF leak if an age-appropriate inactivated or recombinant vaccine cannot be used)
- Receipt of influenza antiviral medication within the previous 48 hours for oseltamivir and zanamivir, previous 5 days for peramivir, and previous 17 days for baloxavir. The interval between influenza antiviral receipt and LAIV4 for which interference might potentially occur might be further prolonged in the presence of medical conditions that delay medication clearance (e.g., renal insufficiency)

Precautions for use of LAIV4 include the following:

- Moderate or severe acute illness with or without fever
- History of GBS within 6 weeks of receipt of any influenza vaccine
- Asthma in persons aged ≥5 years
- Other underlying medical condition (other than those listed under contraindications) that might predispose to complications after wild-type influenza virus infection (e.g., chronic pulmonary, cardiovascular [except isolated hypertension], renal, hepatic, neurologic, hematologic, or metabolic disorders [including diabetes mellitus])

### Recent Influenza Vaccine Licensures

Since the publication of the 2019–20 ACIP influenza vaccine statement, two new influenza vaccines have been licensed: Fluzone High-Dose Quadrivalent (HD-IIV4) and Fluad Quadrivalent (aIIV4). Both new vaccines are expected to be available for the 2020–21 influenza season.

#### Fluzone High-Dose Quadrivalent (HD-IIV4)

In November 2019, FDA licensed Fluzone High-Dose Quadrivalent (HD-IIV4) for persons aged ≥65 years. Like the previously licensed trivalent formulation of Fluzone High-Dose, Fluzone High-Dose Quadrivalent contains 60 *μ*g of HA per vaccine virus, compared with 15 *μ*g per vaccine virus in SD-IIVs (*68*). The dose volume of Fluzone High-Dose Quadrivalent, 0.7 mL per dose, is slightly higher than that for trivalent Fluzone High-Dose (0.5 mL per dose). For the 2020–21 season, Fluzone High-Dose Quadrivalent is expected to replace the trivalent formulation of Fluzone High-Dose.

Immunogenicity and safety of Fluzone High-Dose Quadrivalent were compared with two trivalent formulations of Fluzone High-Dose (each containing one of the two influenza B viruses present in the quadrivalent vaccine) in a randomized trial conducted among 2,670 adults aged ≥65 years. For immunogenicity, Fluzone High-Dose Quadrivalent met prespecified noninferiority criteria compared with trivalent Fluzone High-Dose, with noninferior HA inhibition geometric mean titer ratios and seroconversion rates for all four viruses. For the influenza B viruses, Fluzone High-Dose Quadrivalent met criteria for superior immunogenicity compared with trivalent Fluzone High-Dose that did not contain the same influenza B lineage strain (*68**, **107*). The safety profile of Fluzone High-Dose Quadrivalent was generally similar to that of trivalent Fluzone High-Dose. The prevalence of solicited injection site and some systemic adverse reactions was slightly greater among those who received Fluzone High-Dose Quadrivalent; however, most were mild or moderate, and most resolved within 3 days. Prevalence of serious adverse events (SAEs) was similar among the groups (*68**,**107*).

#### Fluad Quadrivalent (aIIV4)

In February 2020, FDA licensed Fluad Quadrivalent (aIIV4) for persons aged ≥65 years. Like the previously licensed trivalent formulation of Fluad, Fluad Quadrivalent contains the oil-in-water emulsion adjuvant MF59 (*72*). For the 2020–21 season, both Fluad and Fluad Quadrivalent are expected to be available.

Immunogenicity, efficacy, and safety of Fluad Quadrivalent were evaluated in a multicenter randomized controlled trial including 6,790 adults aged ≥65 years who received either Fluad Quadrivalent or a noninfluenza control vaccine (tetanus-diphtheria-acellular pertussis) (*108*). For immunogenicity, Fluad Quadrivalent met prespecified criteria for the proportion of participants achieving serconversion and an HA inhibition titer of 1:40 for all four viruses. Prespecified criteria for efficacy were not met, with an absolute vaccine efficacy of 19.8% (95% CI: −5.27%–38.91%) against influenza confirmed by reverse transcriptase–polymerase chain reaction (RT-PCR). Only seven of the 58 total cases of culture-confirmed influenza that occurred in the Fluad Quadrivalent arm were reported to be due to matched influenza viral strains, making it difficult to draw conclusions concerning efficacy from these data. The prevalence of solicited injection site and systemic adverse reactions was slightly higher among those who received Fluad Quadrivalent than control vaccine; however, most reactions were mild or moderate. Prevalence of SAEs was similar. In a separate immunogenicity and safety study including 1,778 adults aged ≥65 years, Fluad Quadrivalent was compared with two trivalent formulations of Fluad (each containing one of the two influenza B viruses present in the quadrivalent vaccine). For immunogenicity, Fluad Quadrivalent met prespecified noninferiority criteria compared with trivalent Fluad, with noninferior HA inhibition geometric mean titer ratios and seroconversion rates for all four viruses. When considered individually, Fluad Quadrivalent and trivalent Fluad each met criteria for seroconversion and proportion of participants achieving an HA titer of 1:40 for the influenza A(H1N1)pdm09 and A(H3N2) viruses; however, neither the quadrivalent nor trivalent vaccines met these criteria for the influenza B viruses. This might have been related to lower likelihood of generating this response in highly vaccinated population (*108**,**109*). The safety profile of Fluad Quadrivalent was generally similar to that of trivalent Fluad. Solicited injection site adverse reactions were slightly more prevalent among those who received Fluad Quadrivalent; however, most were mild or moderate and resolved within 2–4 days. No differences were noted in the occurrence of systemic adverse reactions or SAEs.

## Storage and Handling of Influenza Vaccines

In all instances, approved manufacturer packaging information should be consulted for authoritative guidance concerning storage and handling of specific influenza vaccines. In general, influenza vaccines should be protected from light and stored at temperatures that are recommended on the package insert. Recommended storage temperatures are generally 36°F–46°F (2°C–8°C) and should be maintained at all times with adequate refrigeration and temperature monitoring. Vaccine that has frozen should be discarded. Specific recommendations for appropriate refrigerators and temperature monitoring equipment can be found in the Vaccine Storage and Handling Toolkit, available at https://www.cdc.gov/vaccines/hcp/admin/storage/toolkit/index.html.

Vaccines should not be used beyond the expiration date on the label. In addition to the expiration date, multidose vials also might have a Beyond Use Date (BUD), which specifies the number of days the vaccine can be kept once first accessed. Once accessed for the first dose, multidose vials should not be used after the BUD. If no BUD is provided, then the listed expiration date is to be used. Multidose vials should be returned to recommended storage conditions between uses. Package information might also specify a maximum number of doses contained in multidose vials (regardless of remaining volume). No more than the specified number of doses should be removed, and any remainder should be discarded. Single-dose vials should not be accessed for more than 1 dose. For information on permissible temperature excursions and other departures from recommended storage and handling conditions that are not discussed in the package labeling, contact the manufacturer.

## Additional Sources of Information Regarding Influenza and Influenza Vaccines

### Influenza Surveillance, Prevention, and Control

Updated information regarding influenza surveillance, detection, prevention, and control is available at https://www.cdc.gov/flu. U.S. surveillance data are updated weekly throughout the year on FluView (https://www.cdc.gov/flu/weekly) and FluView Interactive (https://www.cdc.gov/flu/weekly/fluviewinteractive.htm). In addition, periodic updates regarding influenza are published in *MMWR* (https://www.cdc.gov/mmwr/index.html). Additional information regarding influenza and influenza vaccines can be obtained from CDC-INFO by calling 1-800-232-4636. State and local health departments should be consulted about availability of influenza vaccines, access to vaccination programs, information related to state or local influenza activity, reporting of influenza outbreaks and influenza-related pediatric deaths, and advice concerning outbreak control.

### Vaccine Adverse Event Reporting System

The National Childhood Vaccine Injury Act of 1986 requires health care providers to report any adverse event listed by the vaccine manufacturer as a contraindication to future doses of the vaccine or any adverse event listed in the VAERS Table of Reportable Events Following Vaccination (https://vaers.hhs.gov/docs/VAERS_Table_of_Reportable_Events_Following_Vaccination.pdf) that occurs within the specified period after vaccination. In addition to mandated reporting, health care providers are encouraged to report any clinically significant adverse event after vaccination to VAERS. Information on how to report a vaccine adverse event is available at https://vaers.hhs.gov/index.html.

### National Vaccine Injury Compensation Program

The National Vaccine Injury Compensation Program (VICP), established by the National Childhood Vaccine Injury Act of 1986, as amended, provides a mechanism through which compensation can be paid on behalf of a person determined to have been injured or to have died as a result of receiving a vaccine covered by VICP. The Vaccine Injury Table (https://www.hrsa.gov/sites/default/files/vaccinecompensation/vaccineinjurytable.pdf) lists the vaccines covered by VICP and the associated injuries and conditions (including death) that might receive a legal presumption of causation. If the injury or condition is not in the Table or does not occur within the specified period in the Table, persons must prove that the vaccine caused the injury or condition. Eligibility for compensation is not affected by whether a covered vaccine is used off-label or inconsistently with recommendations.

To be eligible for compensation under VICP, a claim must be filed within 3 years after the first symptom of the vaccine injury. Death claims must be filed within 2 years of the vaccine-related death and not more than 4 years after the start of the first symptom of the vaccine-related injury from which the death occurred. When a new vaccine or a new injury/condition is added to the Table, claims that do not meet the general filing guidelines must be filed within 2 years from the date the vaccine or injury/condition is added to the Table for injuries or deaths that occurred up to 8 years before the Table change (*110*). Persons of all ages who receive a VICP-covered vaccine might be eligible to file a claim. Additional information is available at https://www.hrsa.gov/vaccine-compensation/index.html or by calling 1-800-338-2382.

### Additional Resources

#### ACIP Statements

- General Best Practice Guidelines for Immunization: Best Practices Guidance of the Advisory Committee on Immunization Practices (ACIP): https://www.cdc.gov/vaccines/hcp/acip-recs/general-recs/index.html
- Immunization of Health-Care Personnel: Recommendations of the Advisory Committee on Immunization Practices (ACIP), 2011. MMWR Recomm Rep 2011;60(No. RR-7): https://www.cdc.gov/mmwr/preview/mmwrhtml/rr6007a1.htm
- Recommended Adult Immunization Schedule for Ages 19 Years or Older, United States: https://www.cdc.gov/vaccines/schedules/hcp/adult.html
- Recommended Child and Adolescent Immunization Schedule for Ages 18 Years or Younger, United States: https://www.cdc.gov/vaccines/schedules/hcp/child-adolescent.html

#### Vaccine Information Sheets (VISs)

- VIS for IIV and RIV4: https://www.cdc.gov/vaccines/hcp/vis/vis-statements/flu.pdf
- VIS for LAIV4: https://www.cdc.gov/vaccines/hcp/vis/vis-statements/flulive.pdf

#### Influenza Vaccine Package Inserts

- Trivalent Vaccines: https://www.fda.gov/vaccines-blood-biologics/approved-products/influenza-virus-vaccine-trivalent-types-and-b
- Quadrivalent Vaccines: https://www.fda.gov/vaccines-blood-biologics/approved-products/influenza-virus-vaccine-quadrivalent-types-and-types-b

#### CDC Influenza Antiviral Guidance

- Influenza Antiviral Medications: Summary for Clinicians: https://www.cdc.gov/flu/professionals/antivirals/summary-clinicians.htm

#### Infectious Diseases Society of America (IDSA) Influenza Antiviral Guidance

- Clinical Practice Guidelines by the Infectious Diseases Society of America: 2018 Update on Diagnosis, Treatment, Chemoprophylaxis, and Institutional Outbreak Management of Seasonal Influenza: https://academic.oup.com/cid/article/68/6/e1/5251935

#### American Academy of Pediatrics (AAP) Guidance

- AAP Recommendations for Prevention and Control of Influenza in Children (Red Book Online): https://redbook.solutions.aap.org/ss/influenza-resources.aspx

#### IDSA Guidance for Vaccination of Immunocompromised Hosts

- 2013 IDSA Clinical Practice Guideline for Vaccination of the Immunocompromised Host: https://academic.oup.com/cid/article/58/3/e44/336537

#### American College of Obstetricians and Gynecologists (ACOG)

- Influenza Vaccination During Pregnancy, ACOG Committee Opinion No. 732: https://www.acog.org/Clinical-Guidance-and-Publications/Committee-Opinions/Committee-on-Obstetric-Practice/Influenza-Vaccination-During-Pregnancy
